# Supplementary material for: A Systematic Review of COVID-19 Infection in Kidney Transplant Recipients: A Universal Effort to Preserve Patients’ Lives and Allografts
Source: J Clin Med. 2020 Sep 16;9(9):2986. doi: 10.3390/jcm9092986 (PMC7563559; doi:10.3390/jcm9092986)
Supplement: Supplementary file 1 [file jcm-09-02986-s001.pdf]

Supplementary Item S1. SEARCH STRATEGY.

WEB OF SCIENCE (*n* = 70)

Search History WEB OF SCIENCE (Indexes=SCI-EXPANDED, SSCI, A&HCI, CPCI-S, CPCI-SSH, BKCI-S, BKCI-SSH, ESCI, Timespan=Year to date)		
Set	Results	
# 5	70	(#3 AND #4) AND LANGUAGE: (English)
# 4	3341	(#1 AND #2) AND LANGUAGE: (English)
# 3	15,545	(TS=("COVID 19" OR "Sars Cov2" OR Coronavirus* OR "2019-nCoV" OR "SARS-CoV-2" OR "SARS-CoV")) AND LANGUAGE: (English)
# 2	16,753	(TS=(transplant* OR transplantation)) AND LANGUAGE: (English)
# 1	24,424	(TS=(renal OR Kidney*)) AND LANGUAGE: (English)

PUBMED (*n*=122)

((("COVID 19"[Title/Abstract] OR "Sars Cov2"[Title/Abstract] OR Coronavirus*[Title/Abstract] OR "2019-nCoV"[Title/Abstract] OR "SARS-CoV-2"[Title/Abstract] OR "SARS-CoV"[Title/Abstract]) AND ((renal[Title/Abstract] OR kidney*[Title/Abstract]) AND (transplant*[Title/Abstract] OR transplantation[Title/Abstract]))) AND (recipient*[Title/Abstract] OR patient*[Title/Abstract])) AND eng[la] AND 2020/01/01:2020/07/06[dp]

Search	Query	Results
#10	Search: ((("COVID 19"[Title/Abstract] OR "Sars Cov2"[Title/Abstract] OR Coronavirus*[Title/Abstract] OR "2019-nCoV"[Title/Abstract] OR "SARS-CoV-2"[Title/Abstract] OR "SARS-CoV"[Title/Abstract]) AND ((renal[Title/Abstract] OR kidney*[Title/Abstract]) AND (transplant*[Title/Abstract] OR transplantation[Title/Abstract]))) AND (recipient*[Title/Abstract] OR patient*[Title/Abstract])) AND eng[la] AND 2020/01/01:2020/07/06[dp] Sort by: Most Recent	122

Search	Query	Results
#9	Search: ((("COVID 19"[Title/Abstract] OR "Sars Cov2"[Title/Abstract] OR Coronavirus*[Title/Abstract] OR "2019-nCoV"[Title/Abstract] OR "SARS-CoV-2"[Title/Abstract] OR "SARS-CoV"[Title/Abstract]) AND ((renal[Title/Abstract] OR kidney*[Title/Abstract]) AND (transplant*[Title/Abstract] OR transplantation[Title/Abstract]))) AND (recipient*[Title/Abstract] OR patient*[Title/Abstract])) AND eng[la] Filters: in the last 1 year Sort by: Most Recent	133
#8	Search: ((("COVID 19"[Title/Abstract] OR "Sars Cov2"[Title/Abstract] OR Coronavirus*[Title/Abstract] OR "2019-nCoV"[Title/Abstract] OR "SARS-CoV-2"[Title/Abstract] OR "SARS-CoV"[Title/Abstract]) AND ((renal[Title/Abstract] OR kidney*[Title/Abstract]) AND (transplant*[Title/Abstract] OR transplantation[Title/Abstract]))) AND (recipient*[Title/Abstract] OR patient*[Title/Abstract])) Filters: in the last 1 year Sort by: Most Recent	136
#7	Search: #5 AND #6 Sort by: Most Recent	141
#6	Search: recipient*[Title/Abstract] OR patient*[Title/Abstract] Sort by: Most Recent	6,786,383
#5	Search: #3 AND #4 Sort by: Most Recent	153
#4	Search: #1 AND #2 Sort by: Most Recent	109,566
#3	Search: "COVID 19"[Title/Abstract] OR "Sars Cov2"[Title/Abstract] OR Coronavirus*[Title/Abstract] OR "2019-nCoV"[Title/Abstract] OR "SARS-CoV-2"[Title/Abstract] OR "SARS-CoV"[Title/Abstract] Sort by: Most Recent	43,539
#2	Search: transplant*[Title/Abstract] OR transplantation[Title/Abstract] Sort by: Most Recent	477,052
#1	Search: renal[Title/Abstract] OR kidney*[Title/Abstract] Sort by: Most Recent	880,785

Scopus (*n* = 138)

TITLE-ABS-KEY (renal OR kidney*) AND TITLE-ABS-KEY (transplant* OR transplantation) AND TITLE-ABS-KEY ("COVID 19" OR "Sars Cov2" OR coronavirus* OR "2019-nCoV" OR "SARS-CoV-2" OR "SARS-CoV") AND PUBYEAR = 2020 AND (LIMIT-TO (LANGUAGE , "English"))

Cochrane Database (*n* = 0)

Cochrane Reviews 0	Cochrane Protocols 0	Trials 0	Editorials 0	Special collections 0	Clinical Answers 0	Other Reviews 0

0 Cochrane Reviews matching renal OR kidney* in Title Abstract Keyword AND transplant* OR transplantation in Title Abstract Keyword AND "COVID 19" OR "Sars Cov2" OR coronavirus* OR "2019-nCoV" OR "SARS-CoV-2" OR "SARS-CoV" in Title Abstract Keyword AND recipient* in Title Abstract Keyword - (Word variations have been searched)
Issue 7 of 12, July 2020

Supplementary Item S2. PRISMA Checklist

Section/topic	#	Checklist item	Reported on page #
**TITLE**			
Title	1	Identify the report as a systematic review, meta-analysis, or both.	1
**ABSTRACT**			
Structured summary	2	Provide a structured summary including, as applicable: background; objectives; data sources; study eligibility criteria, participants, and interventions; study appraisal and synthesis methods; results; limitations; conclusions and implications of key findings; systematic review registration number.	1 - 2
**INTRODUCTION**			
Rationale	3	Describe the rationale for the review in the context of what is already known.	2
Objectives	4	Provide an explicit statement of questions being addressed with reference to participants, interventions, comparisons, outcomes, and study design (PICOS).	2-3
**METHODS**			
Protocol and registration	5	Indicate if a review protocol exists, if and where it can be accessed (e.g., Web address), and, if available, provide registration information including registration number.	N/A
Eligibility criteria	6	Specify study characteristics (e.g., PICOS, length of follow-up) and report characteristics (e.g., years considered, language, publication status) used as criteria for eligibility, giving rationale.	3
Information sources	7	Describe all information sources (e.g., databases with dates of coverage, contact with study authors to identify additional studies) in the search and date last searched.	3
Search	8	Present full electronic search strategy for at least one database, including any limits used, such that it could be repeated.	2-3, Suppl. 1

Study selection	9	State the process for selecting studies (i.e., screening, eligibility, included in systematic review, and, if applicable, included in the meta-analysis).	2-3, Figure 1
Data collection process	10	Describe method of data extraction from reports (e.g., piloted forms, independently, in duplicate) and any processes for obtaining and confirming data from investigators.	3-4
Data items	11	List and define all variables for which data were sought (e.g., PICOS, funding sources) and any assumptions and simplifications made.	3-4
Risk of bias in individual studies	12	Describe methods used for assessing risk of bias of individual studies (including specification of whether this was done at the study or outcome level), and how this information is to be used in any data synthesis.	5, Suppl. 3+4
Summary measures	13	State the principal summary measures (e.g., risk ratio, difference in means).	4
Synthesis of results	14	Describe the methods of handling data and combining results of studies, if done, including measures of consistency (e.g., I^2) for each meta-analysis.	4

Section/topic	#	Checklist item	Reported on page #
Risk of bias across studies	15	Specify any assessment of risk of bias that may affect the cumulative evidence (e.g., publication bias, selective reporting within studies).	5, Suppl. 3+4
Additional analyses	16	Describe methods of additional analyses (e.g., sensitivity or subgroup analyses, meta-regression), if done, indicating which were pre-specified.	N/A
**RESULTS**			
Study selection	17	Give numbers of studies screened, assessed for eligibility, and included in the review, with reasons for exclusions at each stage, ideally with a flow diagram.	2-3, Figure 1
Study characteristics	18	For each study, present characteristics for which data were extracted (e.g., study size, PICOS, follow-up period) and provide the citations.	5-6, Tables 1 & 2
Risk of bias within studies	19	Present data on risk of bias of each study and, if available, any outcome level assessment (see item 12).	5, Suppl. 3+4

Results of individual studies	20	For all outcomes considered (benefits or harms), present, for each study: (a) simple summary data for each intervention group (b) effect estimates and confidence intervals, ideally with a forest plot.	8-9, Table 3
Synthesis of results	21	Present results of each meta-analysis done, including confidence intervals and measures of consistency.	N/A
Risk of bias across studies	22	Present results of any assessment of risk of bias across studies (see Item 15).	N/A
Additional analysis	23	Give results of additional analyses, if done (e.g., sensitivity or subgroup analyses, meta-regression [see Item 16]).	9-10, Table 4
**DISCUSSION**			
Summary of evidence	24	Summarize the main findings including the strength of evidence for each main outcome; consider their relevance to key groups (e.g., healthcare providers, users, and policy makers).	10-14
Limitations	25	Discuss limitations at study and outcome level (e.g., risk of bias), and at review-level (e.g., incomplete retrieval of identified research, reporting bias).	13-14
Conclusions	26	Provide a general interpretation of the results in the context of other evidence, and implications for future research.	14
**FUNDING**			
Funding	27	Describe sources of funding for the systematic review and other support (e.g., supply of data); role of funders for the systematic review.	14

Supplementary Item S3. Study Quality Assessment – JBI CRITICAL APPRAISAL CHECKLIST FOR CASE SERIES

STUDIES	Clear criteria for inclusion	Condition measured in a standard, reliable way for all participants	Valid methods for identification of the condition for all participants	Consecutive inclusion of participants	Complete inclusion of participants	Clear report of demographics	Clear report of clinical information	Clear report of outcomes or follow up results of cases	Clear reporting of the presenting site(s)/clinic(s) demographic information	Appropriate statistical analysis	Overall appraisal
[50]Abrishami et al.	Y	Y	Y	U	U	Y	N	Y	Y	Y	High - Included
[3]Akalin et al.	Y	Y	Y	Y	Y	Y	Y	Y	Y	Y	High - Included
[7]Alberici et al.	Y	Y	Y	Y	Y	Y	Y	Y	Y	Y	High - Included
[51]Banerjee et al.	Y	Y	Y	Y	Y	Y	Y	Y	Y	Y	High - Included
[52]Bosch et al.	Y	Y	Y	Y	Y	Y	Y	Y	Y	Y	High - Included
[53]Bossini et al.	Y	Y	Y	Y	Y	U	Y	Y	Y	Y	High - Included
[54]Chen et al.	Y	Y	Y	Y	Y	Y	Y	Y	Y	Y	High - Included
[55]Columbia University	Y	Y	Y	Y	Y	Y	Y	Y	Y	Y	High - Included
[56]Crespo et al.	Y	Y	Y	Y	Y	Y	Y	Y	Y	Y	High - Included

[57]Devresse etal.	Y	Y	Y	U	U	Y	Y	Y	Y	Y	High - Included
[58]Fernandez -Ruizetal.	Y	Y	Y	Y	Y	Y	Y	Y	Y	Y	High - Included
[59]Fung et al.	Y	Y	Y	Y	Y	Y	Y	Y	Y	Y	High - Included
[60]Maritati et al.	Y	Y	Y	Y	Y	Y	Y	Y	Y	Y	High - Included
[61]Mehta et al.	Y	Y	Y	Y	Y	Y	Y	Y	Y	Y	High - Included
[62]Mella et al.	Y	Y	Y	N	N	Y	Y	Y	Y	N/A	High - Included
[63]Montagud -Marrahi et al.	U	Y	U	N	N	N	N	Y	Y	Y	Low - Included
[6]Nair et al.	Y	Y	Y	Y	Y	Y	Y	Y	Y	Y	High - Included
[64]Rodriguez -Cubillo et al.	Y	Y	Y	Y	Y	Y	Y	Y	Y	Y	High - Included
[65]Silva et al.	Y	Y	Y	Y	Y	Y	Y	Y	Y	N/A	High - Included
[66]Trujillo et al.	Y	Y	Y	Y	Y	Y	Y	Y	Y	Y	High - Included
[67]Zhang et al.	Y	Y	Y	Y	Y	Y	Y	Y	Y	N/A	High - Included
[68]Zhu et al.	Y	Y	Y	Y	Y	Y	Y	Y	Y	Y	High - Included

**Y**,**Yes**
**N**, No
**U**, Unclear
**N/A**, Notapplicable

Supplementary Item S4. Study Quality Assessment – JBI CRITICAL APPRAISAL CHECKLIST FOR CASE REPORTS

STUDIES	Clear description of patient's demographic characteristics	Clear description of patient's history and presentation as a timeline	Clear description of the current clinical condition of the patient on presentation	Clear description of diagnostic tests or assessment methods and results	Clear description of intervention(s) or treatment procedure(s)	Clear description of post-intervention clinical condition	Identification and description of adverse events (harms) or unanticipated events	Case report provision of takeaway lessons	Overall appraisal
[9]Akdur et al.	N	N	N	Y	Y	U	N/A	Y	Low - Included
[10]Allam et al.	Y	Y	Y	Y	Y	Y	Y	Y	High - Included
[11]Bartiromo et al.	Y	Y	Y	Y	Y	Y	Y	Y	High - Included
[12]Billah et al.	Y	Y	Y	Y	Y	Y	Y	Y	High - Included
[13]Bussalino et al.	Y	Y	Y	Y	Y	Y	Y	U	High - Included
[14]Chen et al.	Y	Y	Y	Y	Y	Y	Y	Y	High - Included
[15]Cheng et al.	N	N	Y	Y	U	Y	Y	U	High - Included
[16]Chenna et al.	Y	N	Y	Y	N	N	N	U	Low - Included
[17]Dirim et al.	Y	Y	Y	Y	Y	Y	Y	U	High - Included
[18]Fontana et al.	Y	Y	Y	Y	Y	Y	Y	Y	High - Included
[19]Gandolfini et al.	Y	Y	Y	Y	Y	Y	Y	U	High - Included
[20]Guillen et al.	Y	Y	Y	Y	Y	Y	Y	Y	High - Included
[21]Hasan Ahmad et al.	U	U	Y	Y	Y	Y	Y	Y	High - Included
[22]Hsu et al.	Y	Y	Y	Y	Y	Y	Y	Y	High - Included
[23]Huang et al.	Y	Y	Y	Y	Y	Y	Y	Y	High - Included
[24]Jiang et al.	Y	Y	Y	Y	U	Y	Y	Y	High - Included
[25]Kates et al.	Y	Y	Y	Y	Y	Y	Y	Y	High - Included

[26]Kemmner et al.	Y	Y	Y	Y	Y	Y	Y	Y	High - Included
[27]Kim et al.	Y	Y	Y	Y	Y	Y	Y	Y	High - Included
[28]Kocak et al.	Y	Y	Y	Y	Y	Y	Y	Y	High - Included
[29]Kolonko et al.	Y	Y	Y	Y	Y	Y	Y	U	High - Included
[30]Kumar et al.	Y	Y	Y	Y	Y	Y	Y	Y	High - Included
[31]Lauterio et al.	Y	Y	Y	Y	Y	Y	Y	Y	High - Included
[32]Machado et al.	Y	Y	Y	Y	Y	Y	Y	Y	High - Included
[33]Man et al.	N	N	Y	Y	Y	Y	Y	Y	High - Included
[34]Marx et al.	Y	U	Y	Y	Y	Y	Y	U	High - Included
[35]Meziyerh et al.	Y	Y	Y	Y	Y	Y	Y	Y	High - Included
[36]Namazee et al.	Y	Y	Y	U	Y	Y	Y	N	High - Included
[37]Ning et al.	Y	Y	Y	Y	Y	Y	Y	Y	High - Included
[38]Seminari et al.	Y	Y	Y	Y	Y	Y	Y	Y	High - Included
[39]Serrano et al.	Y	Y	Y	Y	Y	Y	Y	Y	High - Included
[40]Shingare et al.	Y	Y	Y	Y	Y	Y	Y	Y	High - Included
[41]SJ Antony et al.	N	U	Y	U	Y	Y	Y	Y	High - Included
[42]Suwanwongse et al.	Y	Y	Y	Y	Y	Y	Y	U	High - Included
[43]Tantisattamo et al.	U	Y	Y	Y	Y	Y	Y	Y	High - Included
[44]Thammathiwat et a	Y	Y	Y	Y	Y	Y	Y	Y	High - Included
[45]Velioglu et al.	Y	Y	Y	Y	Y	Y	Y	Y	High - Included

[46]Wang et al.	**Y**	**Y**	**Y**	**Y**	**Y**	**Y**	**Y**	**Y**	High - Included
[47]Xia et al.	**U**	**Y**	**Y**	**Y**	**Y**	**Y**	**Y**	**Y**	High - Included
[48]Xu et al.	**Y**	**Y**	**Y**	**Y**	**Y**	**Y**	**Y**	**Y**	High - Included
[49]Zhong et al.	**Y**	**Y**	**Y**	**Y**	**Y**	**Y**	**Y**	**Y**	High - Included

**Y,Yes**
**N**, No
**U**, Unclear
**N/A**, Notapplicable
